# Three-Dimensional ISAR Imaging Method for High-Speed Targets in Short-Range Using Impulse Radar Based on SIMO Array

**DOI:** 10.3390/s16030364

**Published:** 2016-03-11

**Authors:** Xinpeng Zhou, Guohua Wei, Siliang Wu, Dawei Wang

**Affiliations:** 1 School of Information and Electronics, Beijing Institute of Technology, Beijing 100081, China; xinpeng_780304@163.com (X.Z.); siliangw@bit.edu.cn (S.W.); primetime2009@126.com (D.W.); 2 Beijing Key Laboratory of Fractional Signals and Systems, Beijing 100081, China

**Keywords:** inverse synthetic aperture radar (ISAR), radar imaging, translational compensation, rotation angle estimation

## Abstract

This paper proposes a three-dimensional inverse synthetic aperture radar (ISAR) imaging method for high-speed targets in short-range using an impulse radar. According to the requirements for high-speed target measurement in short-range, this paper establishes the single-input multiple-output (SIMO) antenna array, and further proposes a missile motion parameter estimation method based on impulse radar. By analyzing the motion geometry relationship of the warhead scattering center after translational compensation, this paper derives the receiving antenna position and the time delay after translational compensation, and thus overcomes the shortcomings of conventional translational compensation methods. By analyzing the motion characteristics of the missile, this paper estimates the missile’s rotation angle and the rotation matrix by establishing a new coordinate system. Simulation results validate the performance of the proposed algorithm.

## 1. Introduction

Three-dimensional imaging is widely used to identify and measure objects [1,2,3,4,5,6,7,8,9], because it provides more objective information, including posture, structure, layout, size, *etc.* A number of three-dimensional ISAR imaging methods are proposed, such as interferometric ISAR imaging methods, imaging methods based on ISAR image sequences, and imaging methods of energy focusing on the time domain. The first two methods are implemented in the frequency domain. The three-dimensional image is reconstructed by calculating the phase difference between the scatterers in ISAR images corresponding to different spatial array antennas in interferometric ISAR imaging methods [10,11]. The three-dimensional structure of the target is reconstructed by acquiring a series of two-dimensional ISAR image sequences and tracking scatterers on ISAR images in imaging methods based on ISAR image sequences [12]. Glint appears when the angle is measured by using the interferometer methods, so higher requirements are put forward to the translational compensation accuracy of echo sequences. High signal processing complexity exists in ISAR imaging methods based on ISAR image sequences.

Due to the curvature of the wavefront in the near-field, the small rotational angle will lead to scatterers’ migration through the resolution cell. The reconstructed image is located away from the rotational center by directly using inverse Fourier, so the three-dimensional ISAR image is obtained through coherent processing of the backscatter field, which is a function of frequencies and rotation angles of the two mutually orthogonal axes [8]. The literature [13] gives a method for short-range 3-D ISAR imaging of the moving target using the interferometric ISAR technique based on a chirp signal system. Methods for imaging of energy focusing on the time domain have been widely used in the near-field [10,11,13,14,15,16]. In these methods, each echo dataset is back projected onto the imaging area. Because each pixel is processed in the imaging area, it has a higher accuracy and avoids any impact from the wavefront curvature and migration through the resolution cell. The large amount of computation that follows makes the imaging method suitable for small target imaging in the near-field.

In the background of miss distance measurement in short-ranges using impulse radar, the dispersion is more significant because the echo spectrum contains a Rayleigh region, a resonance region and an optical area of the target radar cross-section, and the range migration is further deteriorated because its range cell is half the wavelength of the highest frequency, so researchers face the following two challenges. The first is the translational motion compensation method. Translational compensation means that the missile is moved to the closest point of approach (CPA) along the trajectory while maintaining the posture of radar rays to missile. With the requirements of miss distance measurement in short-range, a larger diameter antenna array is established to obtain a high pitch resolution. The missile translational leads to changes in the antenna aperture size, which has an impact on the quality of translational compensation. The existing translational compensation method is not suitable for the scene used in this paper [17,18]. The second is the rotation angle estimation method. Missile rotates around three rotation axes and the rotational angle corresponding to each rotation axis needs to be estimated, which increases the complexity of the algorithm. 

Considering the above challenges, this paper proposes a three-dimensional ISAR imaging method for high-speed targets in short-range based on a single-input multiple-output array. Past research mainly focuses on ISAR imaging in the far-field or the near-field turntable, but pays little attention to ISAR imaging of high-speed targets in short-range using impulse radars. This paper aims to shed light on this latter topic. The outstanding contributions of this paper are the parameter estimation method of the high-speed moving target under a large baseline array, the translational motion compensation method and the rotation angle estimation method in the near field. The remaining sections of this paper are arranged as follows:

Section 2 describes the SIMO-ISAR imaging scene of high-speed moving targets in short-range. The spatial layout of the antenna array is presented.

Section 3 briefly describes the three-dimensional turntable ISAR imaging principle.

Section 4 describes the SIMO-ISAR signal processing chain, emphasizing the motion compensation method and the estimation method of rotation angle. Simulated data are presented to show the performance of these two methods.

Section 5 shows a SIMO-ISAR image using the proposed method in a SIMO-ISAR system. The simulation experiment proves the feasibility of our method in miss distance measurement applications.

The last section gives a conclusion for this paper.

## 2. Results Imaging Scene Description

**Figure 1:**
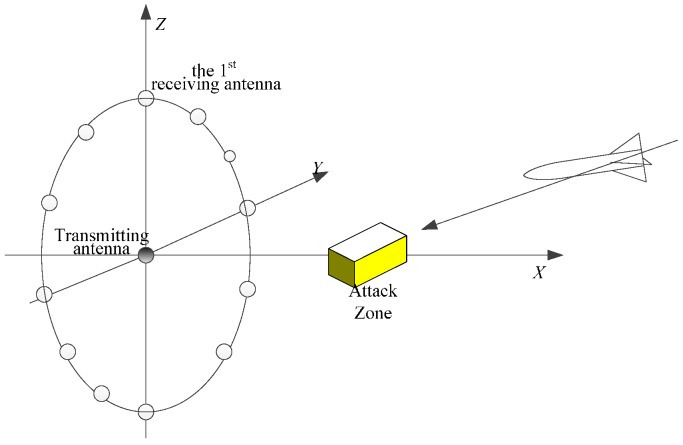
SIMO-SAR scenes of high-speed moving target SIMO-SAR scenes in short-range.

The SIMO-SAR antenna array layout is shown in Figure 1. Transmitting antenna is located at the coordinate origin, 2*k* receiving antennas on the plane *z* = 0 distributed on the circle whose center and radius is the transmitting antenna and *d*, respectively. The first receiving antenna is located at (0, 0, *d*). In a clockwise arrangement, the *k*-th receiving antenna is located at (0, 0, –*d*). The center of attack zone is located in the positive direction of the *x*-axis. The missile motion is an approximately linear uniform motion in the intersection segment. In the SIMO-ISAR imaging system, the first derivative Gauss pulse is transmitted from the transmitting antenna, which is represented as

(1) $$y(t)=A\frac{t-\tau_{0}}{T}\exp\left[-4\pi {\left(\frac{t-\tau_{0}}{T}\right)}^{2}\right]$$

where $A=E_{0}\exp\left[-\frac{1}{16\pi }\right]$, $E_{0}$ is the amplitude, *T* is the effective pulse width, *t* is the time variable, and $\tau_{0}$ is the time delay of the transmitted pulse. The backscatter signal is received by 2*k* receiving antennas, and 2*k* one-dimensional range profiles are obtained. After transmitting *p* pulses, 2*k × p* one-dimensional range profiles are obtained.

## 3. The Three-Dimensional ISAR Imaging Principle

**Figure 2:**
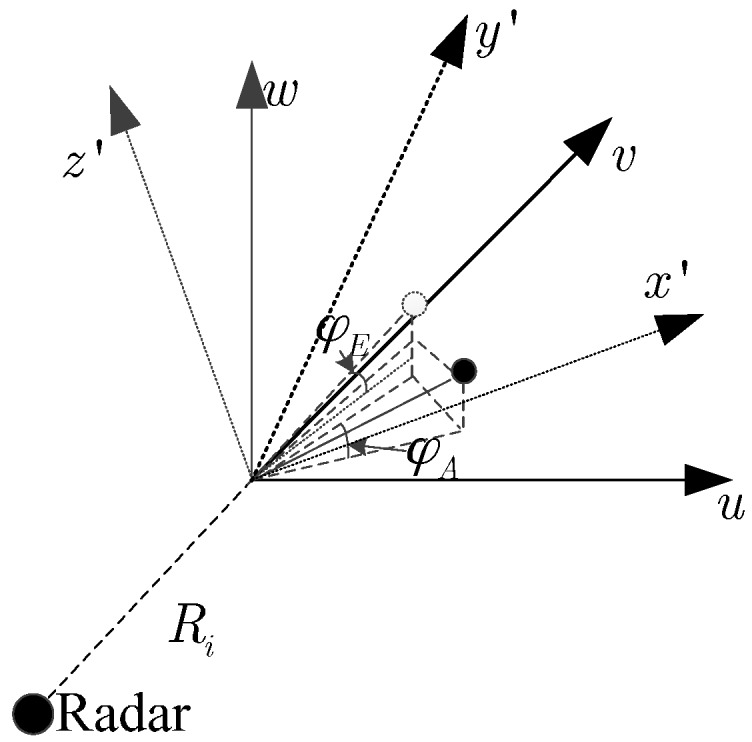
The three-dimensional imaging model of rotating target.

Three-dimensional imaging geometric turntable model is shown in Figure 2. *uvw* coordinate system is a fixed coordinate system, and *x*’*y*’*z*’ coordinate system is a rotating coordinate system.

Let *x*’*y*’*z*’ coordinate system rotates $\varphi_{A}$ and $\varphi_{E}$ around the *z*’-axis and the *y*’-axis, respectively. The relationship between the two coordinate systems *x*’*y*’*z*’ and *uvw* is

(2) $$\left(\begin{array}{c} u\\ v\\ w \end{array}\right)=\left(\begin{array}{ccc} \cos\varphi_{A}\cos\varphi_{E} & -\sin\varphi_{A}\cos\varphi_{E} & \sin\varphi_{E}\\ \sin\varphi_{A} & \cos\varphi_{A} & 0\\ -\cos\varphi_{A}\sin\varphi_{E} & \sin\varphi_{A}\sin\varphi_{E} & \cos\varphi_{E} \end{array}\right)\left(\begin{array}{c} x^{\prime }\\ y^{\prime }\\ z^{\prime } \end{array}\right)$$

When the angle is ($\varphi_{A},\varphi_{E}$), the target scattering function is $f_{\varphi_{A},\varphi_{E}}(u,v,w)$, then the backscatter signal spectrum is represented as

(3) $$\begin{array}{ll} S_{R}(\varphi_{A},\varphi_{E},\xi ) & =\int_{-\infty }^{\infty }s_{R}(\varphi_{A},\varphi_{E},t)e^{-j2\pi \xi t}dt\\ & =\int_{-\infty }^{\infty }\left\{\underset{w,v,u}{∭}f_{\varphi_{A},\varphi_{E}}(u,v,w)s_{T}\left(t-\frac{2R}{c}\right)dudvdw\right\}e^{-j2\pi \xi t}dt\\ & =\underset{w,v,u}{∭}f_{\varphi_{A},\varphi_{E}}(u,v,w)\left\{\int_{-\infty }^{\infty }s_{T}\left(t-\frac{2R}{c}\right)e^{-j2\pi \xi t}dt\right\}dudvdw\\ & =S_{T}(\xi )\underset{w,v,u}{∭}f_{\varphi_{A},\varphi_{E}}(u,v,w)e^{-j2\pi \xi \frac{2R}{c}}dudvdw \end{array}$$

where *R* is the distance between the scatterer (*u*, *v*, *w*) to the radar, as follows:

(4) $$R=\sqrt{{\left(R_{i}+v\right)}^{2}+u^{2}+w^{2}}$$

Substitute Equation (2) into Equation (3):

(5) $$\begin{array}{ll} S_{R}(\varphi_{A},\varphi_{E},\xi ) & =S_{T}(\xi )\underset{z^{\prime },y^{\prime },x^{\prime }}{∭}f_{\varphi_{A},\varphi_{E}}(x^{\prime },y^{\prime },z^{\prime })e^{-j2\pi \xi \frac{R}{c}}dxdydz\\ & =S_{T}(\xi )F(\varphi_{A},\varphi_{E},\xi ) \end{array}$$

Therefore, the inverse Fourier transform of Equation (5) can be written as

(6) $$\begin{array}{ll} f(x^{\prime },y^{\prime },z^{\prime }) & =\underset{\varphi_{A},\varphi_{E},\xi }{∭}F(\varphi_{A},\varphi_{E},\xi )e^{j2\pi \xi \frac{R}{c}}d\varphi_{A}d\varphi_{E}d\xi \\ & =\underset{\varphi_{A},\varphi_{E},k}{∭}F(\varphi_{A},\varphi_{E},\frac{c}{2\pi }k)\left|k\right|e^{j2\pi \xi \frac{R}{c}}dkd\varphi_{A}d\varphi_{E} \end{array}$$

Definitions

(7) $$p(\varphi_{A},\varphi_{E},R)=\int_{-\infty }^{\infty }S_{p}(\varphi_{A},\varphi_{E},\frac{c}{2\pi }k)\left|k\right|e^{j2Rk}dk$$

Equation (7) is an inverse Fourier transform of the product of $S_{p}(\varphi_{A},\varphi_{E},\frac{c}{2\pi }k)$ and |*k*|, the target scattering function can be expressed as

(8) $$f(x^{\prime },y^{\prime },z^{\prime })=\iint_{\varphi_{A},\varphi_{E}}p(\varphi_{A},\varphi_{E},R)d\varphi_{A}d\varphi_{E}$$

## 4. SIMO-ISAR Imaging Processing Chain

Data preprocessing of SIMO-ISAR includes the coherent accumulation, and the removal of clutter and background. SIMO-ISAR data require coherently accumulating the backscattered echoes for further processing. The removal of clutter and background facilitates the detection of the target. In the given scenario, radar backscattering echoes contain not only the useful target echo, but also the clutter and background noise caused by other scatterers outside the target. The existence of clutter and background noise seriously disturbs the target detection capability of the radar and the imaging quality. In order to effectively detect the target and improve the imaging quality, the clutter and background noise should be suppressed or eliminated from the radar backscattering echo. Methods of removing clutter in UWB radar can be referenced in [19,20,21]. Here, we focus on the mathematical derivation and implementation of SIMO-ISAR 3D imaging, so the clutter and the background noise are not addressed in this paper.

### 4.1. Motion Parameter Estimation

At time $t_{p}$, the *i-*th scattering center moves to the position ($x_{ip},y_{ip},z_{ip}$) with velocity *v*, its initial position is ($x_{i0},y_{i0},z_{i0}$), its trajectory unit vector direction is ($l_{x},l_{y},l_{z}$), so the motion equations of the scatterer can be expressed as

(9) $$\begin{array}{l} x_{ip}=l_{x}vt+x_{i0}\\ y_{ip}=l_{y}vt+y_{i0}\\ z_{ip}=l_{z}vt+z_{i0} \end{array}\}$$

The electromagnetic wave travels from the transmitting antenna to the *i*-th scattering centers scattering, and then to the *k*-th receiving antenna. Its distance traveled $R_{k,ip}$ can be written as follows:

(10) $$R_{k,ip}=R_{ip}+\sqrt{{\left(x_{ip}-x_{r,k}\right)}^{2}+{\left(y_{ip}-y_{r,k}\right)}^{2}+{\left(z_{ip}-z_{r,k}\right)}^{2}}$$

where $R_{ip}=\sqrt{x_{ip}^{2}+y_{ip}^{2}+z_{ip}^{2}}$ is the distance between the *i*-th scattering center to the transmitting antenna, that is, the scattering center locates on the ellipsoid whose focuses are the transmitting antenna and the *k*-th receiving antenna.

**Figure 3:**
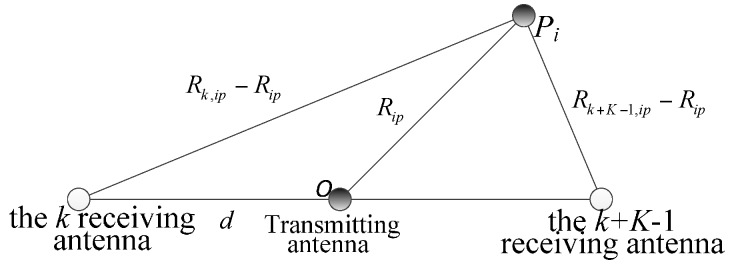
The relationship geometry between the *i*-th scattering center and the *k*-th receiving antenna, the *k + K −* 1 receiving antenna and the transmitting antenna at time $t_{p}$.

The two receive antennas in a straight line with the transmitting antenna and a receiving antenna pair. At time $t_{p}$, a triangle is constructed between the *i*-th scattering centers and any receiving antenna pair, so the transmitting antenna is the midpoint of its base, as shown in Figure 3. For example, scattering centers $P_{i}$, the *k*-th receiving antenna, and the *k + K* − 1 receiving antenna constitute a triangle, then $OP_{i}$ is the triangle midline.

According to Equation (10), the range equation can be obtained corresponding to the receiving antenna *k* + *K* *–* 1:

(11) $$R_{k+K-1,ip}=R_{ip}+\sqrt{{\left(x_{ip}-x_{r,k+K-1}\right)}^{2}+{\left(y_{ip}-y_{r,k+K-1}\right)}^{2}+{\left(z_{ip}-z_{r,k+K-1}\right)}^{2}}$$

$R_{ip}$ is the triangle midline and is obtained according to the geometric relationship, and can be achieved as:

(12) $$4R_{ip}^{2}={\left(R_{k,ip}-R_{ip}\right)}^{2}+{\left(R_{k+K-1,ip}-R_{ip}\right)}^{2}-d_{z}^{2}$$

Solving Equation (12) gives

(13) $$R_{ip}=\frac{R_{k,ip}^{2}+R_{k+K-1,ip}^{2}-2d_{z}^{2}}{2\left(R_{k,ip}+R_{k+K-1,ip}\right)}$$

Substituting Equation (13) into Equation (10), the elliptic equation degenerates to the circle equation whose center is the *k*-th receiving antenna, which can be written as follows:

(14) $$\sqrt{{\left(x_{ip}-x_{r,k}\right)}^{2}+{\left(y_{ip}-y_{r,k}\right)}^{2}+{\left(z_{ip}-z_{r,k}\right)}^{2}}=R_{k,ip}-R_{ip}$$

So

(15) $${\left(x_{ip}-x_{r,k}\right)}^{2}+{\left(y_{ip}-y_{r,k}\right)}^{2}+{\left(z_{ip}-z_{r,k}\right)}^{2}={\left(R_{k,ip}-R_{ip}\right)}^{2}$$

Substitute Equation (9) into Equation (15), simplifying

(16) $$\begin{array}{l} {\left(R_{k,ip}-R_{ip}\right)}^{2}=v^{2}t^{2}+2(l_{x}vx_{i,0}+l_{y}vy_{i,0}+l_{z}vz_{i,0})t\\ -2l_{y}vy_{r,k}t-2l_{z}vz_{r,k}t+D_{i,0}^{2}-2y_{r,k}y_{i,0}-2z_{r,k}z_{i,0}+D_{r,k}^{2} \end{array}$$

where $D_{i,0}^{2}=x_{i,0}^{2}+y_{i,0}^{2}+z_{i,0}^{2}$ and $D_{r,k}^{2}=x_{r,k}^{2}+y_{r,k}^{2}+z_{r,k}^{2}$. Equation (16) can be written as

(17) A•B=C

where $A={\left[\begin{array}{ccccccc} A_{1}^{T} & \vdots & A_{2}^{T} & \vdots & \cdots & \vdots & A_{K}^{T} \end{array}\right]}^{T}$, $B={\left[\begin{array}{ccccccc} v^{2} & l_{x}vx_{i,0}+l_{y}vy_{i,0}+l_{z}vz_{i,0} & l_{y}v & l_{z}v & D_{i,0}^{2} & y_{i,0} & z_{i,0} \end{array}\right]}^{T}$, $C={\left[\begin{array}{ccccccc} C_{1}^{T} & \vdots & C_{2}^{T} & \vdots & \cdots & \vdots & C_{K}^{T} \end{array}\right]}^{T}$. $A_{k}=\left[\begin{array}{ccccccc} \begin{array}{c} t_{0}^{2}\\ t_{1}^{2}\\ \vdots \\ t_{p}^{2}\\ \vdots \\ t_{P-2}^{2}\\ t_{P-1}^{2} \end{array} & \begin{array}{c} 2t_{0}\\ 2t_{1}\\ \vdots \\ 2t_{p}\\ \vdots \\ 2t_{P-2}\\ 2t_{P-1} \end{array} & \begin{array}{c} -2y_{r,k}t_{0}\\ -2y_{r,k}t_{1}\\ \vdots \\ -2y_{r,k}t_{p}\\ \vdots \\ -2y_{r,k}t_{P-2}\\ -2y_{r,k}t_{P-1} \end{array} & \begin{array}{c} -2z_{r,k}t_{0}\\ -2z_{r,k}t_{1}\\ \vdots \\ -2z_{r,k}t_{p}\\ \vdots \\ -2z_{r,k}t_{P-2}\\ -2z_{r,k}t_{P-1} \end{array} & \begin{array}{c} 1\\ 1\\ \vdots \\ 1\\ \vdots \\ 1\\ 1 \end{array} & \begin{array}{c} -2y_{r,k}\\ -2y_{r,k}\\ \vdots \\ -2y_{r,k}\\ \vdots \\ -2y_{r,k}\\ -2y_{r,k} \end{array} & \begin{array}{c} -2z_{r,k}\\ -2z_{r,k}\\ \vdots \\ -2z_{r,k}\\ \vdots \\ -2z_{r,k}\\ -2z_{r,k} \end{array} \end{array}\right]$, $C_{k}=\left[\begin{array}{c} {\left(R_{k,i0}-R_{i0}\right)}^{2}-D_{r,k}^{2}\\ {\left(R_{k,i1}-R_{i1}\right)}^{2}-D_{r,k}^{2}\\ \vdots \\ {\left(R_{k,ip}-R_{ip}\right)}^{2}-D_{r,k}^{2}\\ \vdots \\ {\left(R_{k,i(P-2)}-R_{i(P-2)}\right)}^{2}-D_{r,k}^{2}\\ {\left(R_{k,i(P-1)}-R_{i(P-1)}\right)}^{2}-D_{r,k}^{2} \end{array}\right]$

Matrix **B** can be obtained utilizing the least-squares methods, we can get

(18) $$B={\left(A^{T}A\right)}^{-1}A^{T}C$$

Thereby, the velocity, the motion vector direction and the initial position of the scatterer can be obtained as

(19) $$\begin{array}{l} \hat{v}=\sqrt{B(1)}\\ l_{x}=-\sqrt{B(1)-B{\left(3\right)}^{2}-B{\left(4\right)}^{2}}/\sqrt{B(1)}\\ l_{y}=B(3)/\sqrt{B(1)}\\ l_{z}=B(4)/\sqrt{B(4)}\\ x_{i0}=\sqrt{B(5)-B{\left(6\right)}^{2}-B{\left(7\right)}^{2}} \end{array}\}$$

Trajectory azimuth is

(20) $$\gamma =\arccos\frac{-l_{x}}{\sqrt{l_{x}^{2}+l_{y}^{2}}}$$

Trajectory pitch angle is

(21) $$\beta =\arcsin\frac{-l_{z}}{\sqrt{l_{x}^{2}+l_{y}^{2}+l_{z}^{2}}}$$

Suppose the center of attack zone locates the position $P_{0}$($x_{0},y_{0},z_{0}$), and the CPA position corresponding to the *i*-th scattering center is located at $P_{CPA}$($x_{CPA,i},y_{CPA,i},z_{CPA,i}$), then this straight line $P_{o}P_{CPA}$ is perpendicular to the trajectory of the scattering center and *P_o_* is intersection. According to Equation (9), the $P_{0}$ coordinates can be written as

(22) $$\begin{array}{l} x_{CPA,i}=l_{x}vt_{CPA,i}+x_{i0}\\ y_{CPA,i}=l_{y}vt_{CPA,i}+y_{i0}\\ z_{CPA,i}=l_{z}vt_{CPA,i}+z_{i0} \end{array}\}$$

$t_{CPA,i}$ is the time at which the *i*-th scatterer moves through the CPA. Scalar product between the perpendicular direction vector and the trajectory direction vector can be represented as follows:

(23) $$l_{x}(x_{o}-x_{CPA,i})+l_{y}(y_{o}-y_{CPA,i})+l_{z}(z_{o}-z_{CPA,i})=0$$

Substituting Equation (22) into Equation (13), the CPA time can be attained as:

(24) $$t_{CPA,i}=\frac{l_{x}(x_{o}-x_{0,i})+m_{y}(y_{o}-y_{0,i})+n_{z}(z_{o}-z_{0,i})}{v}$$

Substituting Equation (24) into Equation (22), the *P_o_* coordinates are obtained. Then, the miss distance of the *i*-th scattering center can be achieved as:

(25) $$d_{CPA,i}=\sqrt{{\left(x_{o}-x_{CPA,i}\right)}^{2}+{\left(y_{o}-y_{CPA,i}\right)}^{2}+{\left(z_{o}-z_{CPA,i}\right)}^{2}}$$

### 4.2. Translational Compensation

**Figure 4:**
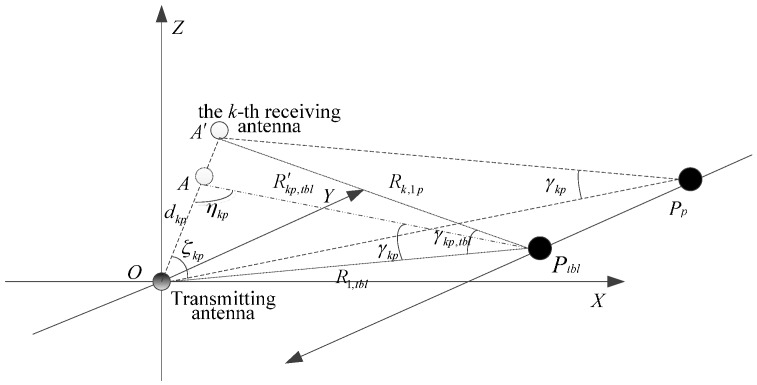
Translational compensation schematic.

Since the warhead scatterer is stable at the scattering center, the warhead scattering point is the equivalent scattering center. As shown in Figure 4, the time the warhead takes to move from the position $P_{p}$ to the position $P_{CPA}$ at time $t_{p}$. at the position $P_{p}$, the angle between the straight lines $OP_{p}$ and $AP_{p}$ is $\gamma_{kp}$, at the position $P_{CPA}$, the angle between the straight lines $OP_{CPA}$ and $A^{\prime }P_{CPA}$ is $\gamma_{kp,CPA}$.

Using the cosine theorem, we can get

(26) $$d^{2}=R_{1p}^{2}+{R^{\prime }}_{k,1p}^{2}-2R_{1p}{R^{\prime }}_{k,1p}\cos\gamma_{kp}$$

Similarly

(27) $$d^{2}=R_{1CPA}^{2}+{R^{\prime }}_{k,1CPA}^{2}-2R_{1CPA}{R^{\prime }}_{k,1CPA}\cos\gamma_{kp,CPA}$$

The angle $\gamma_{kp}$ and $\gamma_{kp,CPA}$ can be obtained by solving Equations (26) and (27).

In order to ensure a constant radar sight angle after translational compensation, at time $t_{p}$, using the sine theorem, the distance $d_{kp}$ between the transmitting antenna to the *k*-th receiving antenna can be attained as,

(28) $$\begin{array}{l} \frac{d_{kp}}{\sin\gamma_{kp}}=\frac{{R^{\prime }}_{kp,CPA}}{\sin\xi_{kp}}=\frac{R_{1,CPA}}{\sin\eta_{kp}}\\ \frac{R_{k,1p}}{\sin\xi_{kp}}=\frac{d}{\sin\gamma_{kp,CPA}} \end{array}\}$$

$R_{kp,CPA}$ and $d_{kp}$ can be obtained by solving Equation (28).

According to the estimated parameters of missile motion, the distance between the receiving antenna and warhead scattering centers can be fitted using Equation (17) as follows:

(29) $$R_{r,kp}=\sqrt{A_{k}B+D_{r,k}^{2}}$$

and the distance $R_{1p}$ between the transmitting antenna and warhead scattering centers. The delay time $\Delta \tau_{kp}$ between pulse sequences received by the *k*-th receive antenna and the $t_{CPA}$ can be represented as follows:

(30) $$\Delta \tau_{kp}=\frac{{R^{\prime }}_{k，1p}+R_{1p}-{R^{\prime }}_{kp,CPA}-{R^{\prime }}_{k,CPA}}{c}$$

At time $t_{p}$, the scattering echo received by the *k*-th receiving antenna can be translationally compensated as [13]:

(31) $$S_{kp,m}(\xi )=S_{kp}(\xi )e^{-j2\pi f\tau_{kp}}F(\varphi_{A},\varphi_{E},k)$$

where $S_{kp}(\zeta )$ and $S_{kp,m}(\zeta )$ is Fourier transform of the scattering echo received by the *k*-th receiving antenna before and after being translationally compensated.

### 4.3. Rotation Angle Estimation

**Figure 5:**
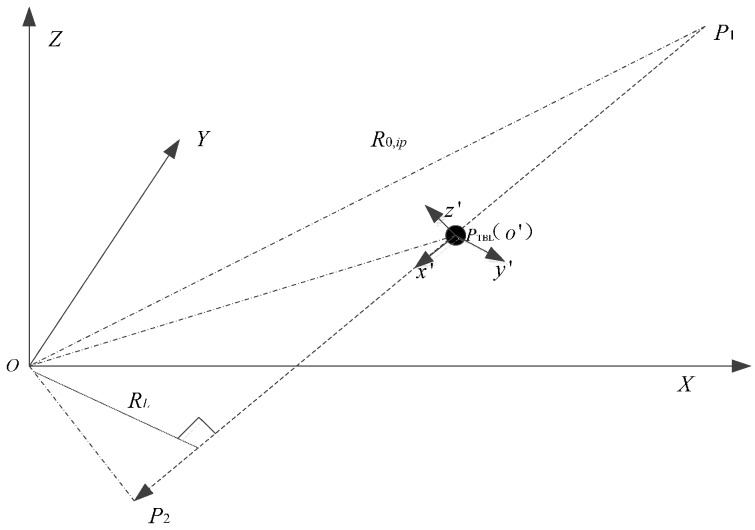
Rotation angle estimation schematic.

The missile rotates around three axes while moving. The warhead coordinates *x*’*y*’*z*’ are established as shown in Figure 5. The coordinate origin is located in the warhead scattering center, the warhead trajectory is the positive direction of the *x*’-axis. The *y*'-axis is located on the *OP*_1_*P*_2_ plane and perpendicular to the warhead trajectory. The *z*’-axis is compliant with the right-hand rule. The missile rotates just around the *z*-axis on the *OP*_1_P_2_ plane.

The minimum distance $R_{L}$ and the distance $R_{p}$ at time $t_{p}$ between the transmitting antenna and the warhead are calculated using Equations (22)–(25) and (30), respectively. Missile rotation angle around the *z*-axis in the *OP*_1_*P*_2_ plane is represented as:

(32) $$\theta_{p}=\{\begin{array}{c} \begin{array}{cc} \arcsin\frac{R_{L}}{R_{p}} & R_{p}\leq R_{p-1} \end{array}\\ \begin{array}{cc} \pi -\arcsin\frac{R_{L}}{R_{p}} & R_{p}>R_{p-1} \end{array} \end{array}$$

### 4.4. Imaging Processing 

The CPA coordinate system *uvw* is established, and its origin is the CPA. The positive direction of the *v*-axis is the direction of radar line of sight, the *u*-axis passes through the CPA on the *OP*_1_*P*_2_-plane and is perpendicular to the *v-*axis. The *w*-axis is compliance with right-hand rule, as shown in Figure 5. After echo translational compensation, the missile rotates around the CPA, and its rotational angle is θ. The relationship between the coordinate system is represented as:

(33) $$\left(\begin{array}{c} u\\ v\\ w\\ 1 \end{array}\right)=R_{z^{\prime }}\;(\theta )\left(\begin{array}{c} x^{\prime }\\ y^{\prime }\\ z^{\prime }\\ 1 \end{array}\right)$$

where $R_{z'}(\theta )$ is the rotation matrix and $R_{z^{\prime }}(\theta )=\left(\begin{array}{cccc} \cos\theta & -\sin\theta & 0 & 0\\ \sin\theta & \cos\theta & 0 & 0\\ 0 & 0 & 1 & 0\\ 0 & 0 & 0 & 1 \end{array}\right)$.

The relationship between the two coordinate systems *xyz* and *uvw* is represented as:

(34) $$\left(\begin{array}{c} x\\ y\\ z\\ 1 \end{array}\right)=R_{z}(\gamma )R_{y}(\beta )T(-x_{tbl},-y_{tbl},-z_{tbl},1)\left(\begin{array}{c} u\\ v\\ w\\ 1 \end{array}\right)$$

where **T** is the translation matrix, $R_{z}(\gamma )$ and $R_{z}(\beta )$ is the transformation matrix, and $T=\left(\begin{array}{cccc} 1 & 0 & 0 & 0\\ 0 & 1 & 0 & 0\\ 0 & 0 & 1 & 0\\ -x_{tbl} & -y_{tbl} & -z_{tbl} & 1 \end{array}\right)$; $R_{z}\left(\gamma \right)=\left(\begin{array}{cccc} \cos\gamma & -\sin\gamma & 0 & 0\\ \sin\gamma & \cos\gamma & 0 & 0\\ 0 & 0 & 1 & 0\\ 0 & 0 & 0 & 1 \end{array}\right)$; $R_{y}\left(\beta \right)=\left(\begin{array}{cccc} \cos\beta & 0 & \sin\beta & 0\\ 0 & 1 & 0 & 0\\ -\sin\beta & 0 & \cos\beta & 0\\ 0 & 0 & 0 & 1 \end{array}\right)$.

Substitute Equation (33) into Equation (34),

(35) $$\left(\begin{array}{c} x\\ y\\ z\\ 1 \end{array}\right)=T^{-1}R_{z}^{-1}(\gamma )R_{y}^{-1}(\beta )R_{z^{\prime }}\left(\theta \right)•R_{z}(\gamma )R_{y}(\beta )T\left(\begin{array}{c} x^{\prime }\\ y^{\prime }\\ z^{\prime }\\ 1 \end{array}\right)$$

(36) $$R\left(\theta \right)=T^{-1}R_{z}^{-1}(\gamma )R_{y}^{-1}(\beta )R_{z^{\prime }}\left(\theta \right)•R_{z}(\gamma )R_{y}(\beta )T$$

R(θ) is the rotation matrix of the SIMO-ISAR system, since the missile rotates only around the z-axis in the coordinate system *x*’*y*’*z*’. Substitute Equation (35) into Equation (6), the missile scattering function $f(x^{\prime },y^{\prime },z^{\prime })$ is reconstructed.

### 4.5. Flow Chart

**Figure 6:**
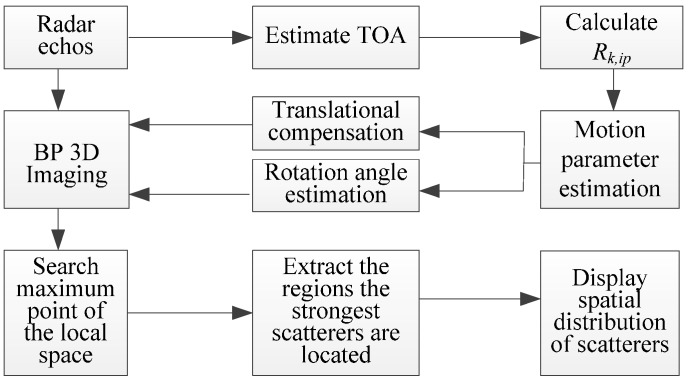
Flow chart of 3D ISAR imaging method proposed in this paper.

The flow chart of the 3D ISAR imaging method of high-speed targets in short-range using an impulse radar based on SIMO array is shown Figure 6.

## 5. Experimental Section

In this section, the proposed 3D SIMO-ISAR imaging method is verified by utilizing simulation data. Simulation data are first used to demonstrate the effectiveness of the first step of the proposed 3D SIMO-ISAR imaging method. The performance of the proposed estimation method is verified by analyzing the estimation accuracy of motion parameters in the white noise environment. The difficult part of the proposed SIMO-ISAR imaging method is achieving the translational compensation. The proposed motion compensation method is validated by providing a fair comparison between the correlation method and the proposed method as well as the analysis of echo sequence before and after motion compensation. Finally, reconstructed 3D ISAR images verify the performance of the SIMO-ISAR imaging system.

**Figure 7:**
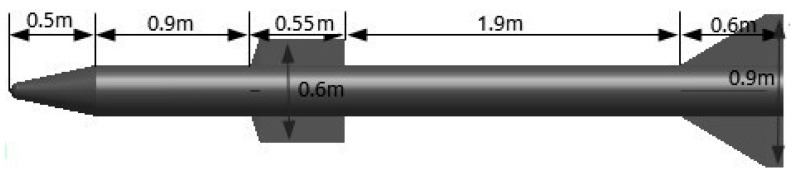
Missile structural model and its size.

The simulation uses the missile model and size shown in Figure 7. Scattering centers are located on the warhead, the tip of the rudder, the tip of the empennage and those intersect discontinuities between the cone and the cylinder, the rudder leading edge and the cylinder, the empennage leading edge and the cylinder. When the edge of the ridge line of the target is irradiated by the electromagnetic wave, edge diffraction is formed and scattering waves are mainly from the target edge diffraction of incident electromagnetic wave [22,23]. Edge diffraction impacts on the focusing energy of the MIMO-ISAR imaging system, but the literature [24] demonstrates that the positions of the scattering centers are basically fixed in the scene set. So, simulation data was collected by an experimental SIMO-ISAR in a spotlight mode. Positions and scattering waveforms of scattering centers are set according to the literature [24].

In the simulation experiment, the SIMO-ISAR imaging system is configured as shown in Figure 1. The radius of the antenna array is 10 m, the number of antenna elements is 8, and the center of attack zone is located at (20 m, 0 m, 0 m). Meanwhile, the waveform parameters of the radar are as follows: the waveform is the 1st Gaussian differential signal in Equation (1), the effective pulse width is 1 ns and the transmitted pulse repetition interval is 1.3 µs, respectively. In our experiment, the missile velocity is 100 m/s, its azimuth angle and the pitch angle is 40° and 4°, respectively. The missile attacks on the attack zone from the point (100 m, 110 m, 10 m) along the linear trajectory, and the flight time is 0.1287 s, acquiring 100,000 pulses with each pulse of 13,000 range bins. Since the width of the transmitted pulse is extremely narrow, the target is almost considered to be static when the pulse is irradiating, so the motion influences of the high-speed target on the echo shape can be ignored [25].

In our simulation test scene, there are no other scatterers except the target and without the speckle phenomenon. To whom may be interested, the clutter and background can be modelled via fractal geometry in the simulation [26,27,28,29]. The model derived from fractal geometry accurately describes the characteristics of clutter and background, and the best simulation data can be sought. The speckle phenomenon arises because the radar backscattered echo of a SAR resolution cell is the coherent sum of the backscattering echo coming from independent discrete scatterers randomly distributed within the resolution cell. In order to adequately describe the speckle phenomenon, the comprehensive physical and statistical characterization of the scatterers is required. SAR speckle modeling is studied in [30,31,32], and the approaches for SAR despeckling are reviewed and a robust framework for performance assessment of SAR despeckling techniques is proposed in [33]. The impact of speckle on the SIMO-ISAR image will be examined in our future research.

### 5.1. Motion Parameter Estimation Result

To analyze the performance of the parameter estimation method proposed in the paper, SNR changes from 10 dB to 30 dB with 2 dB step. Under the different SNR environments, Monte Carlo experiments were conducted 500 times, and the variances of motion parameters are obtained. Table 1 shows that the estimated error is very small, the reason being that all the receiving antenna data is used in the estimation process and the influence of noise is effectively suppressed. It is clearly noted from the table that the estimation performance of motion parameter is improving with the improvement of SNR. When SNR is greater than 16 dB, the estimation performance of motion parameter is significantly improved. In particular, the distance variance is less than 10 cm. When SNR is greater than 24 dB, the distance variance is less than 3 cm.

### 5.2. Translational Compensation Results

**Figure 8:**
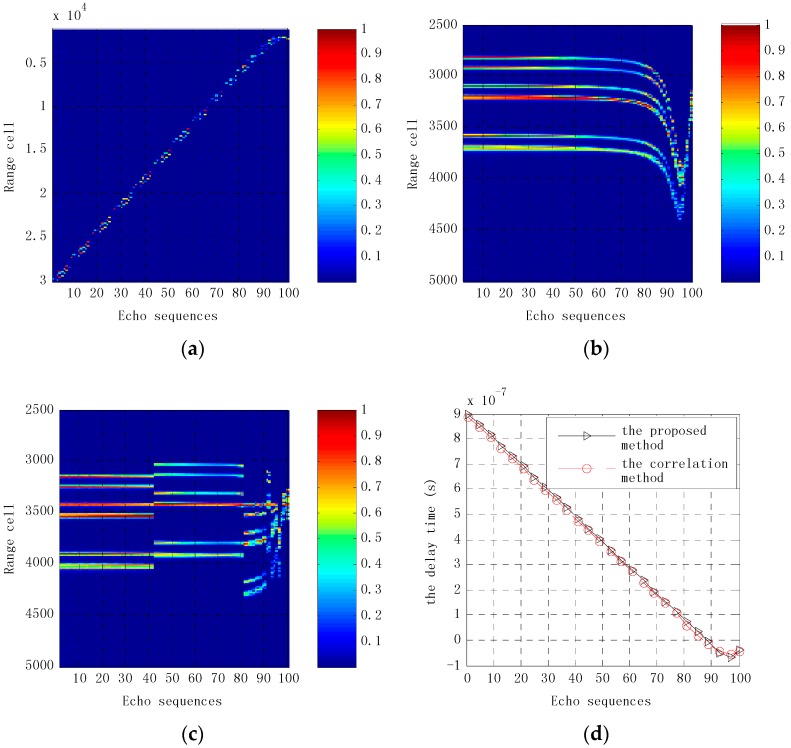
Results of echo sequences received by the first receiving antenna before and after translation compensation. (**a**) Before and after translation compensation; (**b**) After translation compensation using the proposed method in this paper; (**c**) After translation compensation using the correlation method; (**d**) The delay time used in translation compensation.

**Figure 9:**
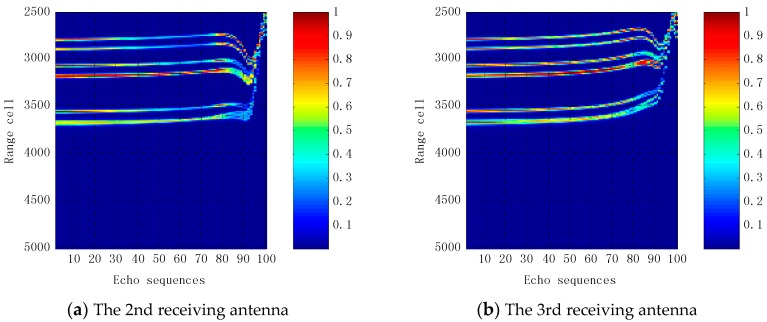

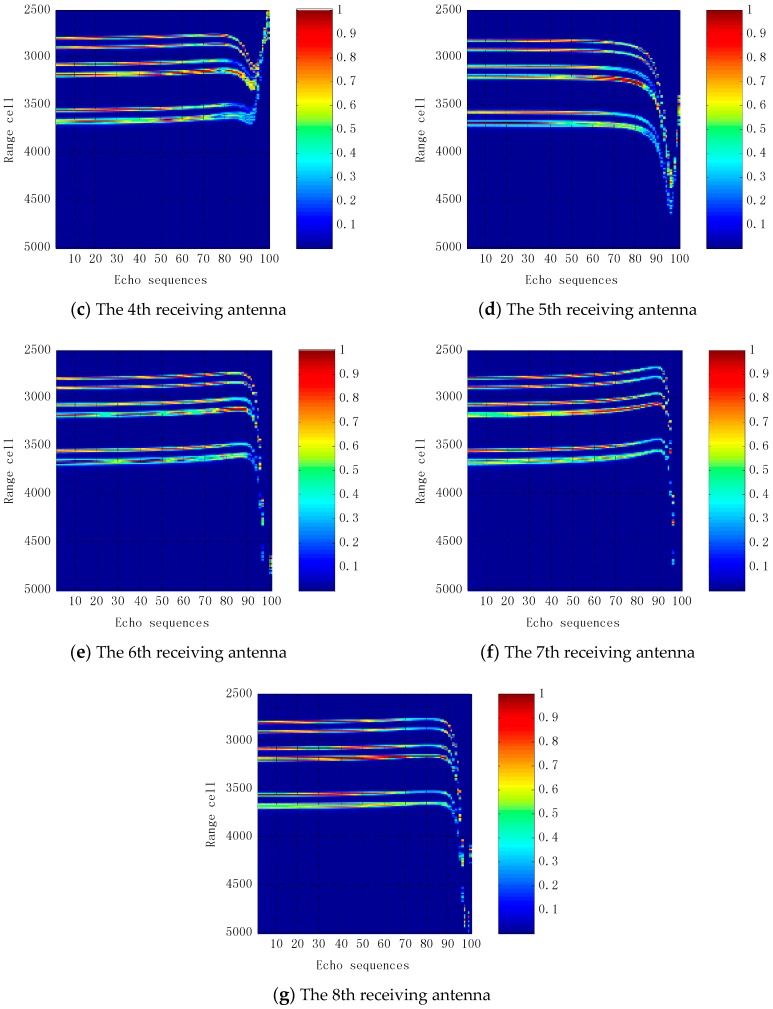
Results of echo sequences received by the 2nd–8th receiving antennas after translational compensation.

Translational compensation is performed on echo sequences using the method proposed in this paper as well as the correlation method [15,16]. Figure 8 presents the results of echo sequences received by the first receiving antenna before and after translation compensation. Before translational compensation, echo sequence positions are approximately quadratically curved, while echoes of the first 80 pulse sequences are in the same cell range after translational compensation using the method proposed in this paper. While scatterers in other echo pulse sequences move gradually away from the antenna, the distance between each of the strong scattering echoes decreases, even being superimposed on each other, so echoes become more complex. This indicates that the missile has a width rotation angle in the near-field of antennas. In the 95th pulse round, it has a strong scattering echo. The reason for this is that the distance between the missile, the transmitting antenna and the receiving antenna is minimal, making it difficult to distinguish each scattering point. Figure 8c shows that echo sequences are clearly divided into three sections after motion compensation using the correlation method, and echo sequences of each segment are not in the same cell range, which impacts the imaging quality. The echoes of the first 41 pulse sequences are in the same range cell after translational compensation, because the missile’s rotation angle is small and the scatterers have barely moved. With the missile movement, its rotation angle increases and the reflection characteristics of the missile are significantly changed, which means the delay time obtained by convolution has significantly changed, as shown in Figure 8d. Figure 8d shows the time delay used in translational compensation. The delay time of the 40th pulse echo sequence is obviously increased. In the 81st echo pulse, the delay time is reduced, and there is a slight time delay fluctuation from the 90th echo pulse to the 100th echo pulse. This is mainly because the missile reflection characteristics are more complex in the antenna near field. Figure 9 shows the results of echo sequence received by other receiving antennas after translational compensation. It can be seen that the first 80 echoes after translational compensation are substantially the same. Comparing Figure 9d and Figure 8b, echo sequences received by the fifth receiving antenna and that by the first receiving antenna are the same after translational compensation. However, echo sequences received by other receiving antennas after translational compensation differ greatly.

### 5.3. Imaging Results

**Figure 10:**
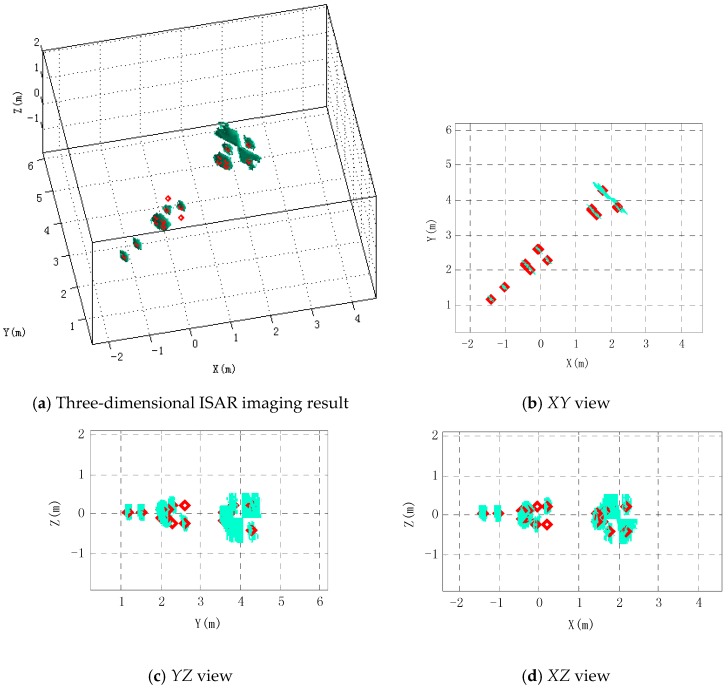
Three-dimensional ISAR imaging result and its three views using the proposed method. Diamonds show the true position of scattering centers.

According to the proposed three-dimensional ISAR imaging method, the missile three-dimensional ISAR image is reconstructed using the proposed three-dimensional ISAR imaging method in this paper in a noiseless environment. As shown in Figure 10, the diamonds represent the actual position of scattering centers whose coordinates are in Table 2. Warhead scattering center and the second scattering center are reconstructed at the actual position. Scattering centers located in the junction between the rudder leading edge and cylinder are indistinguishable from the reconstructed images, because its imaging area covers all junctions. Similarly, scattering centers at the junction between the empennage leading edge and cylinder are indistinguishable. Two scattering centers can be effectively distinguished in the rudder edge and four can be distinguished in the empennage edge. However, there is more energy in the intermediate region of the four scatter points. The 15th and 17th scattering centers are distinguished dependent on the higher range-resolution. It can be seen from the three views of the imaging results that the remaining scattering centers are consistent with their true location except that the 7th scattering point and the 9th scattering point are not reconstructed. 

**Figure 11:**
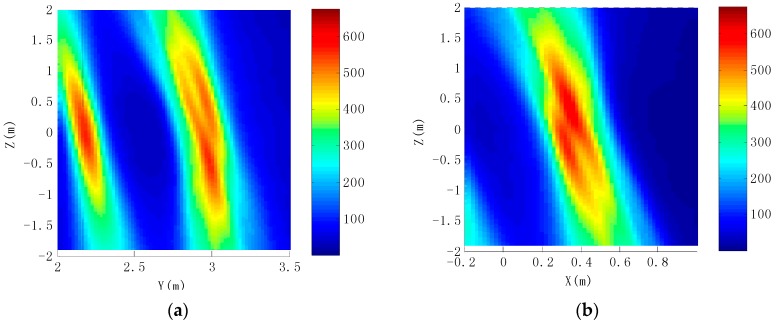

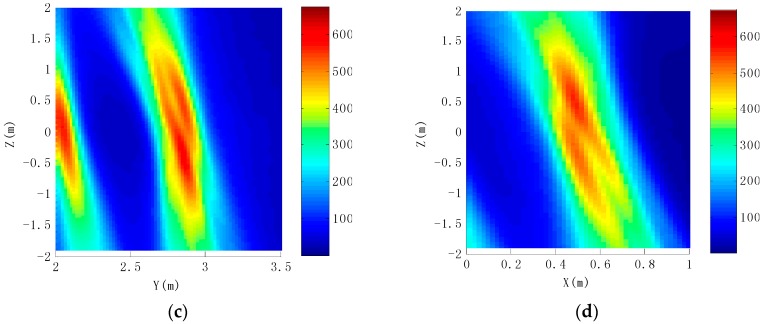
Distribution of scattering centers on rudder edges. (**a**) The section of the 7th and 8th scattering centers in the *YZ* plane; (**b**) The section of the 7th and 8th scattering centers in the *XZ* plane; (**c**) The section of the 9th and 10th scattering centers in the *YZ* plane; (**d**) The section of the 9th and 10th scattering centers in the *XZ* plane.

**Figure 12:**
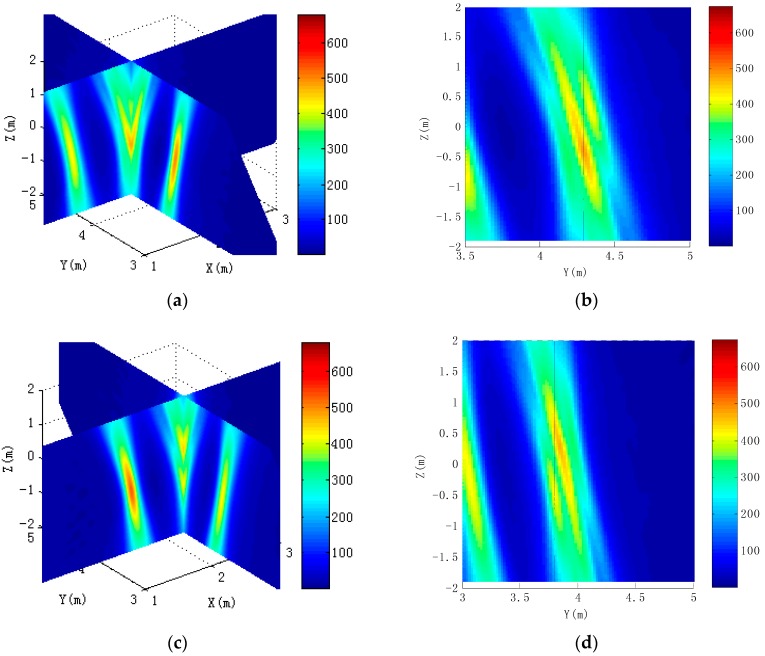
Distribution of scattering centers on empennage trailing edge tips. (**a**) Imaging results of the 15th and 16th scattering centers; (**b**) The section of the 15th and 16th scattering centers in the *XZ* plane; (**c**) Imaging results of the 17th and 18th scattering centers; (**d**) The section of the 17th and 18th scattering centers in the *YZ* plane.

Figure 11 shows that four scattering centers on the rudder edge are distributed on the *YZ* and *XZ* plane, respectively. It can be seen from Figure 11a,b that the 7th and 8th scattering centers are very close to each other on the *Z*-axis, Most of the energy is gathered in the position of the 8th scattering center. Similarly, most of the energy is gathered in the position of the 10th scattering center in Figure 11c,d. The 9th and 10th scattering centers are located in a diagonal position; the distance is large, so they can be effectively distinguished. Because synthetic aperture in the pitch is smaller, the 7th and 8th scattering centers cannot be separated. Similarly, the 9th and the 10th scattering centers are also so. Figure 12 is a profile of scattering centers in the missile empennage tip. It can be seen from Figure 12a,c that the tip of the missile empennage scattering point can be clearly distinguished from the pitch. Its energy is mainly concentrated at the position of the 16th and 18th centers. Figure 12b,d shows that images of scattering centers are similar to the ramp-shaped blade on the *XZ*-plane, also clearly demonstrating the resolution on the pitch.

**Figure 13:**
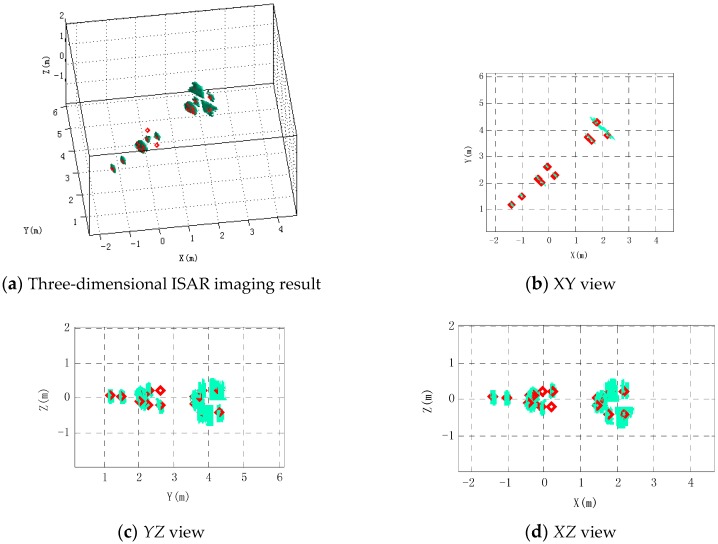
Imaging results when SNR = 15 dB.

**Figure 14:**
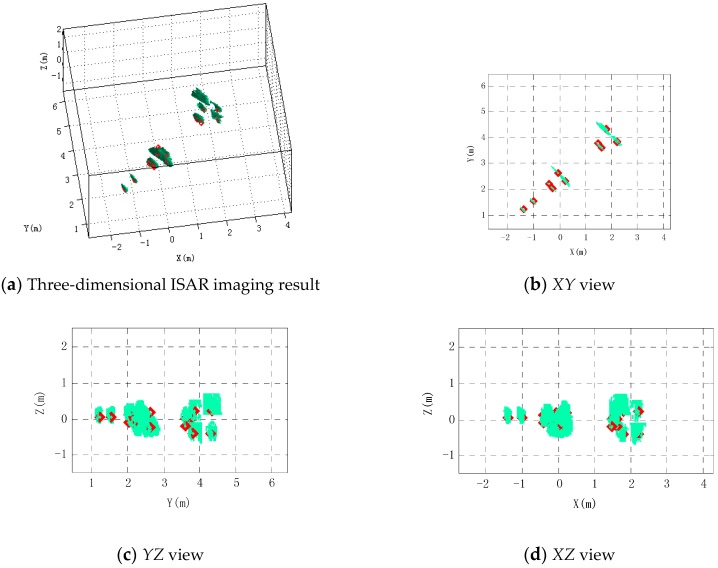
Imaging results when SNR = 10 dB.

Figure 13 and Figure 14 are the imaging results when SNR is 15 dB and 10 dB, respectively. When SNR = 15 dB, each scattering center position is unchanged compared with Figure 10. Four scattering centers in the empennage trailing edge and two scattering centers in the rudder rear edge can be effectively distinguished. When the SNR = 10 dB, scattering centers in the rear edge of the rudder t cannot be distinguished and have come together.

## 6. Conclusions

With the requirements for measuring a high-speed moving target in short-range, a novel translational compensation method and an estimation method using rotation angles for three-dimensional ISAR imaging are proposed. Taking into account the changes of the antenna array aperture in the motion compensation process, the translational motion compensation method proposed compensates the delay time caused by the change in antenna aperture, so the translational compensation performs better. Through the establishment of the warhead coordinates, missile rotation angle is estimated on the plane defined by the missile trajectory and transmitting antenna, and the complexity of the rotation angle estimation is reduced. Simulation results validate the performance of the proposed algorithm. In the following research, we will continue to explore the clutter and background noise impact on SIMO-ISAR imaging and analyze the characteristics of the speckle in SIMO-ISAR images.

## Figures and Tables

**Table 1:** Estimation variance of target motion parameter.

SNR(dB)	$\sigma_{v}$ (m/s)	$\sigma_{l_{x}}$ (×10^−4^)	$\sigma_{l_{y}}$ (×10^−4^)	$\sigma_{l_{z}}$ (×10^−4^)	$\sigma_{l_{CPA}}$ (×10^−2^, m)	$\sigma_{\gamma }$ (×10^−2^, °)	$\sigma_{\beta }$ (×10^−2^, °)
10	1.19	26	22	20	33	19.1	11
12	0.92	16	13	14	19	11.8	8.9
14	0.47	13	10	9.6	14	9.4	5.5
16	0.20	7.8	6.6	6.5	9.1	5.8	3.7
18	0.08	5.6	4.6	4.6	6.6	4.2	2.6
20	0.06	4.7	3.9	3.2	5.5	3.5	1.8
22	0.04	3.2	2.6	2.9	4.2	2.4	1.6
24	0.03	2.3	1.9	2.4	2.9	1.7	1.4
26	0.02	2.1	1.7	1.5	1.9	1.5	0.9
28	0.02	1.9	1.6	1.4	1.5	1.5	0.8
30	0.01	2.0	1.6	1.3	1.4	1.5	0.7

**Table 2:** Position coordinates of scattering centers at $t_{CPA}$ (m).

Scattering Centers *i*	*X*-axis	*Y*-axis	*Z*-axis
1	−1.424	1.186	0.063
2	−1.041	1.508	0.045
3	−0.423	2.165	−0.092
4	−0.418	2.170	0.120
5	−0.281	2.007	0.120
6	−0.287	2.003	−0.092
7	−0.062	2.597	−0.217
8	−0.074	2.282	0.207
9	0.211	2.607	0.207
10	0.199	2.273	−0.217
11	1.457	3.745	0.034
12	1.451	3.582	0.034
13	1.593	3.578	−0.178
14	1.588	3.740	−0.178
15	1.785	4.298	0.225
16	2.195	4.283	−0.411
17	1.768	3.796	−0.411
18	2.178	3.810	0.225
